# Can compensatory culling offset undesirable evolutionary consequences of trophy hunting?

**DOI:** 10.1111/j.1365-2656.2009.01621.x

**Published:** 2010-01

**Authors:** Atle Mysterud, Richard Bischof

**Affiliations:** 1 Centre for Ecological and Evolutionary Synthesis (CEES), Department of Biology, University of OsloP.O. Box 1066 Blindern, NO-0316 Oslo, Norway; 2 Department of Ecology and Natural Resource Management, Norwegian University of Life SciencesP.O. Box 5003, NO-1432 Ås, Norway

**Keywords:** early conditions, evolutionarily enlightened management, large mammals, selective harvesting, sexual ornaments, sexually selected traits, ungulates

## Abstract

1. There is growing concern about the evolutionary consequences of human harvesting on phenotypic trait quality in wild populations. Undesirable consequences are especially likely with trophy hunting because of its strong bias for specific phenotypic trait values, such as large antlers in cervids and horns in bovids. Selective hunting can cause a decline in a trophy trait over time if it is heritable, thereby reducing the long-term sustainability of the activity itself.
2. How can we build a sustainable trophy hunting tradition without the negative trait-altering effects? We used an individual-based model to explore whether selective compensatory culling of ‘low quality’ individuals at an early life stage can facilitate sustainability, as suggested by information from managed game populations in eastern and central Europe. Our model was rooted in empirical data on red deer, where heritability of sexual ornaments has been confirmed and phenotypic quality can be assessed by antler size in individuals as young as 1 year.
3. Simulations showed that targeted culling of low-quality yearlings could counter the selective effects of trophy hunting on the distribution of the affected trait (e.g. antler or horn size) in prime-aged individuals. Assumptions of trait heritability and young-to-adult correlation were essential for compensation, but the model proved robust to various other assumptions and changes to input parameters. The simulation approach allowed us to verify responses as evolutionary changes in trait values rather than short-term consequences of altered age structure, density and viability selection.
4. We conclude that evolutionarily enlightened management may accommodate trophy hunting. This has far reaching implications as income from trophy hunting is often channelled into local conservation efforts and rural economies. As an essential follow-up, we recommend an analysis of the effects of trophy hunting in conjunction with compensatory culling on the phenotypic and underlying genetic variance of the trophy trait.

## Introduction

Accounts of ecosystem change caused by selective human harvesting are accumulating (Allendorf & Hard 2009). In terrestrial ecosystems, traits targeted by trophy hunters are often sexually selected traits that evolved as signals of superior phenotypic quality. Antlers in cervids and horns in bovids are the most common targets of trophy hunters. The best supported evidence of evolutionary consequences of trophy hunting comes from wild sheep (Coltman *et al.* 2003; Garel *et al.* 2007), where trophy hunting decreased size and altered shape of horns. Long-term reduction in average trophy trait values undermines the sustainability of the activity itself.

How to manage populations that are evolving has become a key focus in fisheries (Jørgensen *et al.* 2007; Kuparinen & Merilä 2008) and to an increasing degree in terrestrial ecosystems (Allendorf *et al.* 2008; Coltman 2008). There are few viable suggestions on how trophy hunting can be made ‘evolutionarily enlightened’ (Ashley *et al.* 2003). Reducing offtake of trophy antlers will lower the chance or increase the time it takes to get an evolutionary response (Thelen 1991; Lindsey, Roulet & Romañach 2007). Allowing prime-aged individuals to breed for a few years before being shot may suffice to maintain their superiority as breeders. However, long delays may often be undesirable due to the cost of waiting and the risk that the animal will die before harvest; a risk that increases substantially after prime-age. One could argue that economic income from trophy hunting should not be an important motivator for accommodating high harvest quotas. Yet these activities may benefit the local economy by providing employment and using local services. Furthermore, trophy hunting income is sometimes channelled into conservation efforts (Lewis & Alpert 1997; Lindsey *et al.* 2006). Is it possible to counter the selection pressure induced by trophy hunting by means other than reducing the overall harvest?

**Fig. 1:**
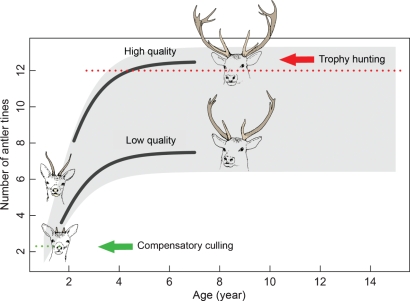
Graphical representation of the concept to use compensatory culling at an early age (aimed at yearling males of low quality) to offset the undesirable effects of trophy hunting of prime-aged individuals (aimed at males with large antlers). Antler size is a trait indicative of phenotypic quality; it is highly variable and clearly visible from a distance already in yearlings, thus permitting selective harvesting of low quality individuals. The grey area signifies the range of trait values assumed during growth by individuals with different growth potentials. The dotted red line is drawn at the lower threshold for trophy hunting (12 antler tines), whereas the dotted green line marks the upper threshold for compensatory culling (variable).

One possible practice is the so-called ‘Wahlabschuβ’ (i.e. selective shooting) commonly used in countries such as Germany, Poland and Hungary for sustainable management of red deer (*Cervus elaphus* L.) and other ungulate populations. This Germanic tradition, called compensatory culling from here on, entails the selective culling of individuals that show poor antler characteristics (e.g. based on tine length or volume) at an early age (Fig. 1, Lockow 1991; Drechsler 1992). Compensatory culling operates on the premise that early age antler sizes correlate with later age antler sizes (Bartos, Bahbouh & Vach 2007). The larger yearlings are allowed to mature to be available for trophy hunting at a later stage. The hunting of low-quality males is performed by local hunters (Mysterud, Tryjanowski & Panek 2006). The compensatory culling is their opportunity to hunt and acquire meat (S. Csanyi, pers. comm.). Compensatory culling does not have to be costly and is therefore a feasible management alternative. Similar approaches involving compensatory culling have also been suggested for ungulate management in Spain (Torres-Porras, Carranza & Pérez-González 2009) and the USA (Williams, Krueger & Harmel 1994), although certain assumptions that the strategy is based on are still debated (Koerth & Kroll 2008).

There is a noticeable absence of any theoretical evaluation of whether compensatory culling at an early life stage can counter the undesirable effects of trophy hunting (Fig. 1). It may seem intuitive that appropriately applied selective pressures can alter the fitness landscape in a way that may accommodate trophy hunting while at the same time minimizing the decline in trait quality in the population. However, there are a number of factors involved besides young–adult correlation in traits and heritability, including population dynamic responses to selective culling at different ontogenetic stages, the impact of age structure changes and density-dependent effects on individual trait values (Kruuk *et al.* 2002; Mysterud *et al.* 2005). These complexities demand a more elaborate approach than just intuition in order to generate valid predictions about the feasibility of compensatory culling. Here we develop an individual-based model of a male red deer population to explore the potential short- and intermediate-term effects (up to 100 years) of compensatory culling. The model was parameterized and validated using the extensive empirical knowledge available primarily from red deer populations in Norway and Scotland. This includes information of population dynamics and how trophy traits, age, density and breeding success are related.

## Materials and methods

We used an individual-based simulation model of a male red deer population, where age and antler size were individual- and time-dependent attributes. We follow Grimm *et al.* (2006) guidelines for describing individual-based models. Assumptions and the model parameter space are listed in Table 1, while the validation is given in the Supporting Information. Our model allows trait values that were associated with greater fitness to emerge under a given selection regime, and is thus akin to optimization approaches (e.g. Cody 1974; Smith 1978; Rice 2004), albeit in our case non-deterministic. Although simplified, the model was based on a complex system with the following important challenges:

**Table 1:** Overview of input parameters and assumptions of the individual-based model of male red deer population and antler size dynamics

Parameter, assumption	Symbol	Sources	Value(s) used
Demography			
Natural mortality	*S*_*0,a*_	Catchpole *et al.* (2004); Coulson *et al.* (2004)	**Nonlinear function of age**
Density-dependent survival	*S*_*a*_	Catchpole *et al.* (2004)	{**true**, false}
Reproductive rate (male calvesborn per female)	*f*	Coulson *et al.* (2004); Langvatn *et al.* (2004)	**0·32** (constant)
Male:female ratio at birth		Clutton-Brock *et al.* (2002)	**1**
Female population size	*N*_f_		**400** (constant)
Male carrying capacity	*K*		**300**
Constant natural abioticenvironment			**True**
Antler size (*x*_*a,i*_)			
Heritability	*h*^2^	Kruuk *et al.* (2002)	{0, 0·15, **0·329**, 0·7}
Permanent individual error	*ε*_p_	Kruuk *et al.* (2002)	{0·1, 0·15, **0·3**}
Annual individual error	*ε*_*a*_	Lockow (1991)	{**0·1**, 0·15, 0·2}
Density-dependent antler growth	*x*_*a*,*i*_(*N*/*K*)	Kruuk *et al.* (2002); Mysterud *et al.* (2005)	**{true**, false}
Mutation rate	*μ*	Radwan (2008)	**0·01**
Antler growth curve parameters	*P*, *B*_2_, *B*_3_	Mysterud *et al.* (2005)	Nonlinear model fit to data **9·86, 4·44, 0·35**
Relative annual breeding success	ABS	Kruuk *et al.* (2002)	**Linear**
Management			
Overall hunting quota (proportionof male population size)	*Q*		**0·15**
Culling risk (proportion of poorquality individuals)	*q*_c_	Predictor	0 to 0·9
Culling threshold	*τ*_c_	Predictor	1·5 to 3
Trophy pressure (number of trophies)	*h*_t_	Predictor	{0, 3, **5**, 8}
Trophy threshold	*τ*_t_		**12**

Terms in ‘{}’ represent alternative values for which simulations were run. Default values (i.e. those best supported by empirical evidence or conjecture) are shown in bold. Boolean responses (true/false) are used where appropriate.

1. overlapping generations;
2. age-dependent survival and reproductive rates;
3. stochasticity in population dynamics and trait expression;
4. different selection pressures (magnitude and direction) on an ontogenetically changing trait at various ages;
5. additional effects on the focal trait through density dependence.

### Overview

#### Purpose

The purpose of the model was to explore whether compensatory culling at the yearling stage could compensate for the trait-depressing effects of trophy hunting at the adult stage.

#### State variables and scales

Four scales were considered in the model: (i) individual male red deer; (ii) birth cohort; (iii) the population; and (iv) the environment (defined by the management regime). Individuals were described by the following state variables: age, individual trait size potential, expressed and age-specific antler size. Individuals 5- to 8-years old were considered prime-aged. Cohorts were characterized by the average potential size of all individuals that had entered the cohort at the time of birth. The population was described by its size, i.e. the total number of males alive at a given time. The abiotic environment was assumed to be constant and the model was not spatially explicit. Only males were modelled explicitly. The female segment of the population was assumed to be of fixed size and total recruitment dependent only on female fecundity. Maternal effects on offspring trait values were ignored.

#### Process overview and scheduling

**Fig. 2:**
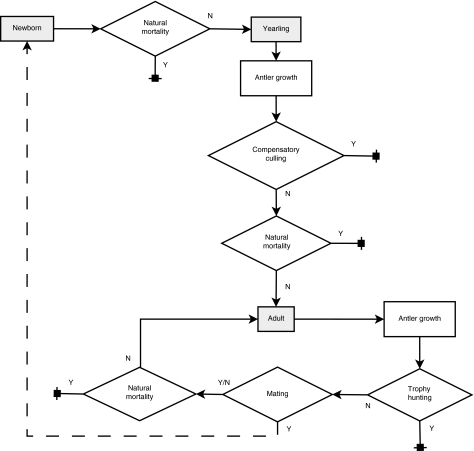
Life history of male red deer in the individual-based model showing age classes (grey-shaded boxes), processes and survival transitions. The hashed line connecting reproducing adults with newborns indicates the contribution of adult males to next year’s newborns. Deaths are marked with small black boxes with a strikethrough.

Simulations followed an annual schedule of events (Fig. 2), which began with calving, followed by antler growth of individuals 1 year and older, hunting mortality, mating (i.e. recruitment allocation to individual males) and finally natural mortality (i.e. winter and spring mortality). Although the rut and mating often coincide with the hunting season, for simplicity, we modelled hunting and mating sequentially. Similarly the winter/spring mortality overlaps with calving and early ontogeny of newborns, but these events were separated in the model. At the end of each model cycle, survivors aged 1 year and the cycle began anew with the calving season (Fig. 2).

### Design concepts

Population dynamics and dynamics of the focal trait in the population emerged as a result of individual characteristics. All interactions among individuals were modelled implicitly.

Survival and relative breeding success were interpreted as probabilities. Furthermore, stochastic elements were included in trait inheritance (as the error term in the sire-son regression) and trait expression (as the annual individual error).

#### Observation

For model testing, the distribution of sizes for each age class was observed annually. For model analysis, we recorded population-level variables (mean trait value, age distribution, population size, number of trophy-sized individuals) over the entire simulation period (or at designated assessment times), and individual-level variables (all state variables) in the final year of the simulation.

### Details

#### Initialization

The initial population age structure was of limited importance. As long as there was a sufficient number of individuals that survived and reproduced during the first few years of the simulation, the population would go to an equilibrium age structure within a few years. Potential antler sizes in the initial population were drawn from a uniform distribution between 0 and 20 antler tines.

#### Input

We modelled three types of management regimes: (i) unbiased hunting mortality followed by natural mortality; (ii) trophy-biased hunting followed by natural mortality; and (iii) compensatory culling and trophy-biased hunting followed by natural mortality. The input parameters that guided the implementation of hunting, such as harvest pressures and selection thresholds, are summarized in Table 1.

#### Submodels

##### M1. Antler growth

The focal trait was antler size, measured in number of antler tines. We assumed that growth of antlers followed the Gompertz growth curve (Kruuk *et al.* 2002). The addition of a normally distributed annual error *ε*_*a*_ (Fig. S1; with SD expressed in per cent of size potential at age *a*) leads to: 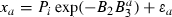 eqn 1

The parameters *B*_2_ (4·4) and *B*_3_ (0·4) were estimated by fitting the Gompertz growth curve to red deer antler tine and age (*a*) data from Norway (Mysterud *et al.* 2005) using nonlinear regression (Fig. S6). *P*_*i*_ is the individual growth potential (i.e. growth curve asymptote), with an average of 9·9 antler tines estimated from the Norwegian red deer data. We approximated density-dependent antler growth as a linear function of male population density (*N*/*K*, newborns excluded) with a slope of −1. Due to the small variation in the number of antler tines of yearlings (most individuals have 2), selection at age 1 was based on size (length) of the spikes. To enable use of the same unit for the trait under selection in both yearlings and adults, we assumed that spike length translates into (non-observable) tine number (used here as a continuous trait).

##### M2. Heritability

The asymptotic (potential adult) antler size of individuals entering the simulation as newborns was calculated based on their father’s size potential by using the sire-offspring regression where the slope of the regression equals 1/2 heritability (*h*^2^) and adding a normally distributed random error (Table 1), analogous to the permanent environment error described by Kruuk *et al.* (2002). To account for overlapping generations and age structure, the assigned asymptotic trait values of all individual’s sired by the members of a cohort were regressed towards the mean trait value in their fathers’ birth cohort at the time of that cohort’s birth (i.e. before viability and fecundity selection acted on the fathers’ cohort).

**Fig. 4:**
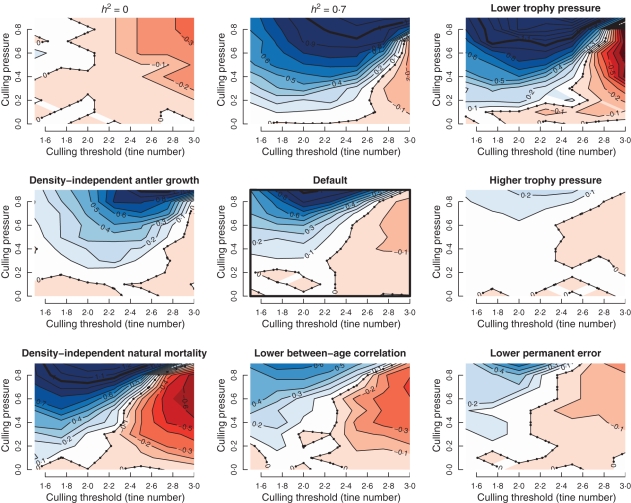
Contour plots of compensation in the number of trophies as a function of culling threshold and culling pressure. The centre plot represents predictions resulting from default model settings (Table 1). The remaining panels illustrate the consequences of various violations of assumptions or deviations from default parameter values (clockwise starting top left corner): (i) no heritability of antler size; (ii) higher heritability (*h*^2^ = 0·7); (iii) reduced trophy pressure (3 trophy-specific tags per year); (iv) elevated trophy pressure (5 trophy tags per year); (v) reduced permanent error (*ε*_p_ = 0·15 × 9·9 antler tines); (vi) increased annual error (*ε*_*a*_* =*0·2**×**current size potential); (vii) density-independent natural mortality; and (viii) density-independent antler growth.

Heritability of antler mass in the most detailed study of red deer was 0·33 (Kruuk *et al.* 2002), and their review of other deer studies points to estimates usually in the same range though extremes include heritability estimates from 0·09 to 0·75. We assume a similar range for antler tines. Heritability of size of horns seems to be similar (0·24 in bighorn sheep *Ovis canadensis* Shaw; Poissant *et al.* 2008). We accounted for variation in empirical reports by testing model predictions under different heritability settings (Table 1, Fig. 4). The approach outlined above ignores genetic progress in the female part of the population. Although female preference for males with larger antlers was a basic tenant of the model, we assumed that mating was non-assortative, i.e. good males do not selectively mate with good females and vice versa.

Variation in the model was maintained through the permanent individual error introduced in each individual’s potential size and subsequent regression of offspring trait value on the father’s potential size. This was an artificial way of maintaining genetic variation, but note that it was intended only as a proxy for the underlying true processes.

We allowed for a mutation rate of 0·01, termed a realistic mutation rate/genome/generation for sexual ornaments (Radwan 2008). Individuals affected by mutation received a trait value between 0 and 20, picked randomly from a uniform distribution. Mutations were not required to avoid trait fixation in our model, because variation was maintained through the sire/offspring regression as described above. Instead, mutations were permitted in order to facilitate the stochastic appearance (and disappearance) of extreme trait values, within the limits provided (0–20 in our model).

##### M3. Mating (and relative breeding) success

We assumed that an adult male’s annual relative breeding success (ABS, interpreted here as its share in the number of 0-year-olds at the beginning of the next time step) was a function of its antler mass *m* and age *a* (no negative ABS values were allowed), such that: 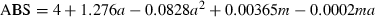 eqn 2

We adjusted the regression coefficient associated with the mass:age interaction to avoid eventual negative effects of mass on ABS at higher ages as arose if we used the coefficient provided by Kruuk *et al.* (2002). We also added an intercept to give ABS values similar to those reported in Kruuk *et al.* (2002). Because the coefficients estimated by Kruuk *et al.* (2002) were for antler size expressed in mass, we converted antler tine number into mass using a logistic function (Fig. S2) fit to data from Iberian red deer provided kindly by Y. Fierro and summarized in Fierro *et al.* (2002).

##### M4. Hunting mortality

Hunting mortality risk was implemented based on an annual harvest quota, calculated as a fixed proportion *Q* of current male population size (*N*). Depending on the current management regime, harvest was determined as follows:

1. Without trophy hunting, the entire annual harvest *H* was unbiased with respect to age and antler size, and constituted proportion *Q* of the male population.
2. With trophy hunting, a fixed number of trophy ‘tags’*h*_t_ out of the total annual harvest quota were designated to be filled with individuals with antler sizes above the trophy threshold *τ*_t_, regardless of age. The remainder of the quota was filled without considering age and antler size. Thus, the number of individuals left for unbiased harvest (*h*_ub_) was calculated as: 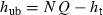 eqn 3
3. Adjustments to trophy pressure were accomplished by altering the number of designated trophy tags without changing the total number of animals harvested.
4. Compensatory culling followed a step function, affecting (with probability *q*_c_) only individuals who were 1-year old and with antler sizes below the culling threshold *τ*_c_.

Compensatory culling was not incorporated in the total harvest quota, hence its immediate effects in terms of survival were additive. Some compensation (not to be confused with compensatory culling) can be expected due to the density-dependent effects on natural mortality, but such compensation is likely weak (Lebreton 2005). Hunting and natural mortality were also assumed to be additive. We make the simplifying assumption of no effect of overall male quality on female fecundity (i.e. annual breeding success was relative), and we ignore other potential indirect effects of selective harvesting as they are usually weak (Mysterud, Coulson & Stenseth 2002) unless sex ratios are extreme (Milner-Gulland *et al.* 2003).

##### M5. Recruitment and population growth

Assuming an age structure similar to that reported for Norwegian red deer (Langvatn & Loison 1999), and annual age-specific female survival rates following a pattern and magnitude reported for red deer from the Isle of Rum, Scotland (Catchpole *et al.* 2004), we calculated the number of newborn males (assuming equal sex ratio at birth) produced each year as 0·32 times the number of females. The population was stable because the female population size and reproductive rate were fixed. In Norwegian red deer, there is weak density dependence in reproductive rates that affect only primiparous females (Langvatn *et al.* 2004), so we chose not to model this. Although we did not include density-dependent effects on fecundity in the model, recruitment of adults was density dependent due to density-dependent survival of newborns and yearlings.

##### M6. Natural mortality

We defined natural mortality risk experienced by individuals during simulation as a function of age (Fig. S4; Catchpole *et al.* 2004), with high mortality at the youngest ages, a rapid decrease in mortality as individuals approach prime age, low mortality throughout prime age and a senescent effect beginning around the age of 9 (Fig. S3) by using: 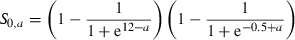 eqn 4

 We incorporated density-dependent effects on survival for newborns, yearlings and individuals older than 8 years (Fig. S4; Catchpole *et al.* 2004) by formulating their natural mortality function as (see also Collier & Krementz 2007): 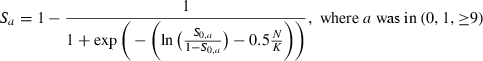 eqn 5

Because the evidence for a relationship between antler size and natural mortality in ungulates is inconclusive (Bonenfant *et al.* 2009), we did not account for it in the model.

### Simulations and assessment of uncertainty

**Fig. 5:**
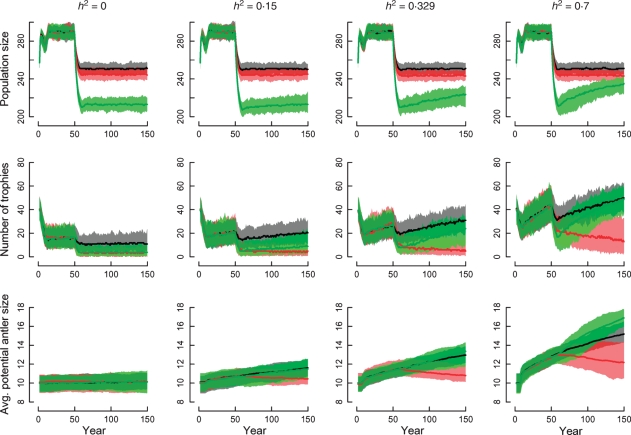
Time series of population size, number of trophy-sized individuals and antler size potential over cohorts for three different settings of heritability (*h*^2^). Solid lines represent point-wise median values from 100 simulations for each parameter setting and equivalently coloured semi-transparent areas show the associated 95% confidence bands (point-wise quantiles). Responses in the absence of trophy-biased hunting are shown in black, responses to trophy-biased hunting without compensation are shown in red, and responses to trophy-biased hunting with compensatory culling are shown in green. The first 50 years of each simulation proceeded without hunting.

We explored the behaviour of the model and made predictions by running 50 simulations over 150 years for each set of parameters. The first 50 years of each simulation were implemented without any hunting mortality, allowing population size and age structure to reach their respective equilibria. Because we incorporated a more biologically realistic mechanism for trait inheritance than typical optimization approaches, we used a fixed time limit instead of waiting for trait values to approach an asymptote. The rationale was that, under selection, heritability stays near its original level for a limited time only (5–10 generations, sometimes more, Falconer & Mackay 1996). We therefore provided forecasts only for the short and intermediate term (up to 100 years). Preliminary simulations indicated that stable age distribution and equilibrium population size were achieved within just a few years (see also Fig. 5).

The main responses measured were the effect of parameter changes on (i) the distribution of potential and expressed antler sizes and (ii) the number of trophy-sized (≥12 antler tines) individuals. We also assessed various other attributes of the population to monitor the population dynamic response to and impact on compensation. Responses were assessed in the 10th, 25th, 50th and 100th year of each simulation, approximately equivalent to 2, 5, 10 and 20 times red deer generation time (∼5 years, Mysterud, Yoccoz & Langvatn 2009). We report median values of the response parameters from all simulations associated with a given parameter set. Upper and lower CI limits around each median were calculated as the 0·975 and 0·025 quantiles of the distribution of the response parameter, respectively. Growth model fitting, individual-based simulations and analysis were conducted in r 2.8.0 (R Development Core Team 2008).

## Results

### Effects of compensatory culling

**Fig. 3:**
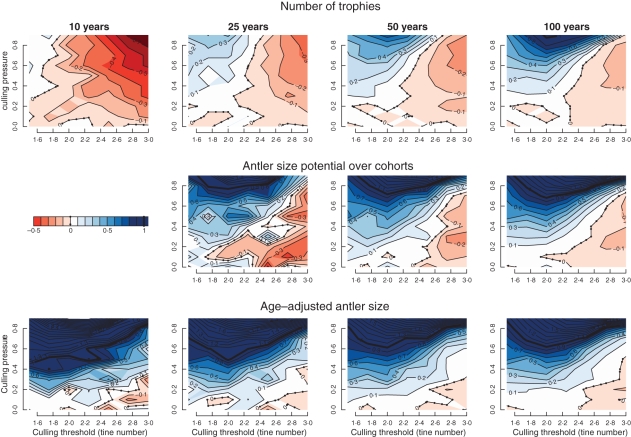
Contour plots showing the simulated effects of culling pressure and culling threshold on compensation in (i) the number of trophy-sized individuals; (ii) the median antler size potential over cohorts (to monitor evolutionary change in trait values); and (iii) the age-adjusted antler size of red deer older than 2 years. Full compensation (solid thick black line) is achieved when compensatory culling leads to levels of the response equal to the level attained without trophy-biased hunting, whereas 0 compensation (hashed thick black line) is determined by the response level for trophy-biased hunting in the absence of compensatory culling. Shades of red indicate negative compensation, shades of blue positive compensation.

Results of the assessment of model validity are detailed in the Supporting Information (Figs S5-S7). Simulations show that selective culling of poorer quality individuals at a young age can compensate for the detrimental effect of trophy hunting on both the mean value of the trait under selection and on the absolute number of prime-aged individuals that were trophy quality (i.e. ≥12 antler tines, Fig. 3). Numerical compensation was substantially weaker than the change in the mean trait value, because the latter was partially attained through reduction in overall population size. Both culling pressure and culling threshold were crucial for the outcome of compensatory culling in both the short and intermediate term. Reaching even moderate levels of compensation required high culling pressures. With certain parameter settings, compensatory culling can be counterproductive. Most or all yearlings fall below the culling threshold if it was set too high, decreasing the number of trophies instead of facilitating compensation. This was especially apparent during the early years of compensatory culling, when trait values in the population have not yet had sufficient time to respond to selection. In general, higher thresholds (but within the bulk of the yearling size distribution) and stronger pressures yielded greater compensation once trait values had responded to this form of selection.

### Role of trophy hunting pressure

Selective culling of ‘poor quality’ yearlings increases the total number of trophy quality individuals in the population but full compensation was only achieved at relatively low trophy hunting pressure. Increasing trophy pressure quickly diminished the effectiveness of compensatory culling. With default parameter settings (Table 1), raising the number of annual trophy tags from 5 to 8 individuals reduced the maximum attainable level of compensation in the number of trophy quality individuals from just above 100% to between 20% and 30% (Fig. 4).

### Short-term effects

Hunting affects population age structure and, as such, also the distribution of any trait that is age-dependent. We therefore compared observed antler sizes and age-corrected antler sizes among adult individuals (≥2 years), but found no obvious age structure effects in terms of the patterns of compensation in response to selective culling. Possible reasons for this were (i) as culling removes only yearlings, it does not change the relative size of the different age classes after the yearling age; and (ii) age structure was assessed before culling and trophy hunting, hence immediate selective effects (same year) were not detected.

To distinguish between viability selection and selection that results in an evolutionary shift in average trait values, we needed a measure of trait values in the population that was not sensitive to short-term effects of differential persistence of individuals with certain trait values. We obtained such a measure by first calculating the average antler size potential of each cohort at that cohort’s birth (i.e. before selection operated on that cohort). In any given year, the median antler size potential over cohorts is then calculated as the median of the average cohort-specific antler sizes of all cohorts still represented in the population.

We compared the effect of compensatory culling on the median antler size potential over cohorts (based on cohorts at birth), with that on the average age-adjusted number of antler tines (based on individuals). Changes in the former should represent an evolutionary response, although the latter would also include the short-term effect of differential longevity. However, we found little indication of a contribution of persistence effects to the observed patterns of compensation (Fig. 3).

### Violating assumptions and changing parameters

#### Heritability

Raising heritability from 0·329 to 0·7 increased the efficiency of compensatory culling due to a better link between selectivity and genetic response (Figs 4 and 5). At the same time, it increased the amount of compensation needed because the effect of trophy hunting on the trait distribution was also more severe (Fig. 5). When we assumed no heritability (*h*^*2*^ = 0), the effects of compensatory culling on antler size were negative for most of the range of culling thresholds, and neutral at low culling thresholds, the latter presumably due to density-dependent effects on antler growth (Fig. 4). Heritability also influenced the demographics of the population (Fig. 5).

#### Yearling-to-adult correlation

As expected, the amount of annual variation in individual antler sizes affected the outcome of management. Increasing this variation led to a decrease in correlation between yearling and adult antler sizes, which in turn reduced the efficiency of compensatory culling with respect to the number of trophy-sized individuals available for hunting (Fig. 4).

#### Density-dependent antler growth

By definition, annual harvest quota (trophy hunt + unbiased harvest) remained unchanged, regardless of the amount of trophy pressure, and the removal of yearlings below the culling threshold appeared not to cause a gain in antler sizes due to further release from density dependence. However, density dependence caused a shift in optimal culling thresholds (a shift in the blue area from right to left in Fig. 4), because expressed trait values were naturally lower with than without density dependence.

#### Density-dependent survival

Removing the assumption of density-dependent natural survival amplifies the compensatory effect of selective culling, whereas the overall pattern remained unchanged qualitatively (Fig. 4). Negative density-dependent survival buffers population responses to management, as factors that increase population size also decrease natural survival, whereas factors that cause a decline in the population increase natural survival. Consequently, density-dependent natural survival buffers the effects of compensatory culling.

#### Permanent error in antler size

The error added to each individuals antler size potential was the source of both genetic variation (at least in part), as well as the permanent environmental error (equivalent to the one estimated by Kruuk *et al.* 2002). A more thorough explanation of its role and rationale is provided in the Supporting Information. We found that reducing this error from a standard deviation of 0·3 × *P* (*P*= average size asymptote in Norwegian red deer, not to be confused with individual antler size potential *P*_*i*_) to 0·15 × *P* resulted in a diminished effectiveness of compensatory culling (Fig. 4). As the variation in the trait potential was reduced, selection had a smaller range of values to operate on, resulting in smaller responses to selection. This in turn caused a reduction in the speed of the trait changes, and consequently the change in the number of trophy-sized individuals.

## Discussion

Our theoretical work showed that, within the bounds of a series of empirically well-supported assumptions, compensatory culling of poor-quality individuals at an early life stage had the potential to at least partially compensate for the trait-altering effects of trophy hunting in the short and intermediate term (up to 100 years).

### Compensation and trophy hunting pressure

Large males in natural populations sire a disproportionate number of offspring. Trophy hunting may in extreme cases cause a reversed relationship with smaller males siring more offspring, as reported in bighorn sheep (Coltman *et al.* 2003). This depends on the harvest pressure. The large-sized elephant (*Loxodonta africana* Blumenbach) bulls in Tarangire national park, Tanzania, retained a higher mating success even under poaching pressure (Ishengoma *et al.* 2008). Our model suggests compensatory culling might be enough to offset the likelihood for directional selection induced by trophy hunting. The average trait in the population responded readily to even mild compensatory culling in our simulations (Fig. 3). However, to achieve noticeable compensation in the number of trophy-sized individuals (≥12 tines) required substantial compensatory culling pressures. Achieving full compensation in terms of the number of trophies available may require average antler size among prime-aged individuals to be driven up substantially higher than it would be even under natural (non-hunting) conditions. Though empirical evidence for a cost of bearing antlers and horns is weak (Bonenfant *et al.* 2009), it is likely that fitness costs at very high antler sizes make such over-compensation in antler sizes difficult or perhaps impossible to achieve. It seems that the best results one can expect from compensatory culling will be achieved when trophy hunting pressures are relatively low (*c*. 10% in our model for the default parameter setting).

### Short-term effects

Observed change in a focal trait may not necessarily be due to direct (fecundity) selection on the trait, but a result of changes to population structure and viability selection (Vaupel & Yashin 1985). Such effects are clearly less enduring than an actual change in the genetic make-up of the population. In the early stages of compensatory culling (<25 years in our model), a temporary decline in the number of trophies appeared even with low trophy hunting pressures (Fig. 3). This was likely so because average trait values have to first increase in the population, before a sufficient number of high-quality yearlings fall above the threshold to form the next generations of prime adults.

The extent of the initial negative compensation was likely dependent on the culling threshold and on the strength of negative density dependence on antler growth. We expected a greater degree of compensation with negative density-dependent antler growth than with density-independent antler growth. However, changes to the age structure and short-term viability selection did not contribute noticeably to the observed changes in the trait distribution (Fig. 4). Though surprising, it could be explained if one considers that without density dependence, culling thresholds may be set higher, because yearling trait values will on average be higher than in the presence of density dependence. Consequently, gains in antler sizes through density-dependent effects may be offset by the decrease in overall effectiveness of selective culling due to lower optimal thresholds. This was consistent with the finding that the addition of density-dependent effects on antler growth shifts areas with positive compensation (blue area in Fig. 4) towards lower culling thresholds. We note that different assumptions about the hunting regime (e.g. a reduction in the overall harvest quota) may result in a positive effect of density-dependent antler growth on compensation through selective culling. Such short-term effects may be stronger in other systems depending on life histories or if vital rates and population size are assessed at different times during the year than in our model.

### Heritability

The most important assumptions for compensatory culling to work are heritability of the focal trait and young–adult correlation in trait values. Heritability of antler quality has received support not only from quantitative genetics studies (Kruuk *et al.* 2002), but also several alleles for antler growth in red deer have been identified (Hartl *et al.* 1995). Indeed, changes in allele frequency have been linked to selective trophy harvesting (Hartl *et al.* 1991; Hartl, Zachos & Nadlinger 2003). Greater heritability of the focal trait means that, in theory, a stronger and faster response can be expected to compensatory culling. However, greater heritability also means a stronger response to trophy hunting in the first place, requiring greater compensation. Indeed, increased heritability resulted in stronger effects of compensatory culling (Fig. 4). Heritability (for any given simulation run) was fixed in our model. Heritability may depend on environmental conditions (Lynch & Walsh 1998). As a consequence, we probably overestimated the speed with which antler size evolved in response to fecundity selection (breeding success) and viability selection (selective harvesting). Expected changes in heritability are generally not large and the response to selection can be maintained over many generations (Falconer & Mackay 1996).

To make our model tractable, fecundity selection acted on a single trait (in combination with age) and viability selection was purely age-specific. Natural selection may act on many different and often correlated characters simultaneously (Lande 1982; Law 1991). A lack of evolution in antler mass of red deer on Rum, Scotland, in the face of heritability and selection could be explained with environmental covariance between the focal trait (antler mass) and some unmeasured trait (e.g. body condition; Kruuk *et al.* 2002). These are important considerations, and the ability to detect and account for them is one of the benefits of quantitative genetics over optimization approaches traditionally used in ecological studies of evolution (Lande 1982; Lynch & Walsh 1998).

### Young–adult trait correlation

The concept of compensatory culling depends on the predictability of adult trait values from trait values at a young age. Although this has been shown to be the case in several field studies (e.g. Schmidt *et al.* 2001), there are also contradictory reports (Drechsler 1992; Koerth & Kroll 2008). However, sample sizes in these studies are rather low. The most thorough study followed 51 male red deer ageing (Bartos *et al.* 2007). The correlation between antler traits (mass, length, tine number) decreased with increasing distance between ages, but it remained relatively high for antler tine number even at age distances of 6 years. The correlation between individual antler tine numbers at age 3 and 8 years was 0·78, whereas the correlation between adjacent ages ranged between 0·68 and 0·84 (Bartos *et al.* 2007). In red deer on Rum, Scotland, antler length in yearlings and the number of antler points as 2-year-olds were also well correlated (*r* = 0·67, Schmidt *et al.* 2001). Antler composition and development are strongly dependent on early conditions and feeding (Landete-Castillejos, García & Gallego 2007), and the gene:environment interaction may prove important to determine the correlation between antler trait at young and old age. Only further empirical research can determine under which conditions this critical assumption holds.

### Model limitations

Trait evolution in real ungulate populations is complicated by the existence of alternate mating strategies (Hogg 1984), which we did not consider in our model. Yearling and subadult males allocate very little in antlers relative to body weight compared to at the prime-age stage (Vanpé*et al.* 2007). During the senescent stage, there is evidence of alternative antler growth tactics. Larger males continue to allocate heavily to antlers, whereas smaller males lower allocations (Vanpé*et al.* 2007). It was assumed that the best males were able to continue defending a mating territory or a harem, whereas the smaller males were forced to alternative mating tactics with little benefit of antlers. Similarly, we made only the asymptote of the Gompertz model individual-dependent; the other growth curve parameters were constant. However, it is possible that not only the size potential but also the speed with which it is attained varies (e.g. Lockow 1991). Violating this assumption could have important consequences, for example if selection would favour individuals with a lower asymptote but faster growth. This strategy would protect an individual from compensatory culling as a yearling, and subsequently protect it from trophy hunting as an adult.

Because we calculated offspring phenotype based on a regression on sire trait values instead of modelling genes, our model does not properly account for the loss in genetic variation due to strong directional or stabilizing selection as a result of selective hunting. Whereas traits under sexual selection generally have high amounts of additive genetic variation (review in Radwan 2008), the addition of strong viability selection through biased harvesting is bound to reduce not only expressed variability in trait values but also variability in their genetic basis (Bulmer 1971; Shnol & Kondrashov 1993). A more mechanistic and realistic approach to trait inheritance is required to evaluate the effects of selective harvesting on genetic variation in antler sizes in red deer and other ungulates. For now, our model assumes that, regardless of the selectivity path taken, there remains sufficient variation in the trait for selection to operate on. An important requirement for sustainability of trophy hunting with the help of compensatory culling in the long-run is the maintenance of variation in the trait and its genetic basis in the face of increased levels of stabilizing selection. We therefore consider the investigation of the dynamics of additional moments of the trait distribution following the implementation of a more mechanistic genetic model an essential and logical next step.

Several other important aspects of trophy hunting and compensatory culling remained unexplored, including (i) the effects of environmental stochasticity in recruitment; (ii) the possibility that the relationship between antler size and reproductive success may not be linear; and (iii) alternate hunting systems and different temporal patterns of harvest and culling. It also remains an open question whether compensatory culling can work with other sexually selected traits desired by trophy hunters such as mane size and colour in male lions (Whitman *et al.* 2004). Also, for large carnivores, one may run into conservation issues if implementing compensatory culling that is increasing overall mortality and thus reducing markedly population size (Fig. 5).

## Conclusion

Although heritability and young–adult correlation in the focal trait were required for compensatory culling to work, shifting parameters and violating several assumptions revealed model resilience in terms of qualitative predictions. So far, there has been no formal statistical analysis of antler size trends in the cultures practising compensatory culling. However, one piece of empirical evidence for the utility of the approach comes from red deer trophy collections preserved in the castle of Detmold in Lippe, North Rhine-Westfalia, Germany. Specimens in the collection date from the end of the 17th to the beginning of the 19th century. Despite the fact that red deer antlers have been the most sought-after trophies for centuries in this culture, comparisons with red deer data in the same area today give no indication that trophy sizes have decreased notably over time (Ueckermann 1990). Although this evidence is somewhat anecdotal and awaiting more thorough analysis, it is contrasted by cases of hunter-caused trophy trait regressions in Africa (von Brandis & Reilly 2008), North America (Coltman *et al.* 2003) and in western Europe (Garel *et al.* 2007) without a tradition of compensatory culling. We conclude that evolutionarily enlightened harvesting (Ashley *et al.* 2003; Gordon, Hester & Festa-Bianchet 2004) and trophy hunting may not be incompatible, and that compensatory culling has the potential to make trophy hunting sustainable.

## Supporting Information

**Fig. S1.** Simulated sizes for individuals with three different antler size potentials (grey: 12 antler tines, red: average size asymptote in Norwegian red deer, blue: 8 antler tines) and two different levels of the annual errors in growth: (a) SD = 10% of potential size at a given age, (b) SD = 20% of potential size at a given age.

**Fig. S2.** Logistic function fit (solid black line) to antler mass and tine number data from Iberian red deer, kindly provided by Y. Fierro and summarized in Fierro *et al.* (2002).

**Fig. S3.** Survival probability (natural) plotted as a function of age as used in the simulation model of male red deer.

**Fig. S4.** Different levels of simulated age-specific survival probability (natural) of male red deer.

**Fig. S5.** Twenty-five-year dynamics of a simulated population of male red deer without hunting.

**Fig. S6.** Boxplots showing the distribution of antler sizes over all ages in Norwegian red deer (left) and a simulated population from the individual-based model. Both the real and the simulated population were hunted.

**Fig. S7.** Distribution of correlation estimates between simulated antler tine numbers of yearling and 6-year-old red deer, when the annual error (*ε*) in antler growth was set to 0·1 (thin hashed line), 0·15 (thick solid line) and 0·2 (thin solid line).

As a service to our authors and readers, this journal provides supporting information supplied by the authors. Such materials may be re-organized for online delivery, but are not copy-edited or typeset. Technical support issues arising from supporting information (other than missing files) should be addressed to the authors.
