# Supplementary material for: Can compensatory culling offset undesirable evolutionary consequences of trophy hunting?
Source: J Anim Ecol. 2010 Jan;79(1):148–60. doi: 10.1111/j.1365-2656.2009.01621.x (PMC2810430; doi:10.1111/j.1365-2656.2009.01621.x)
Supplement: Supplementary file 1 [file jae0079-0148-SD1.doc]

**Supporting Information for “Can compensatory culling offset undesirable evolutionary consequences of trophy hunting?”** by Mysterud and Bischof


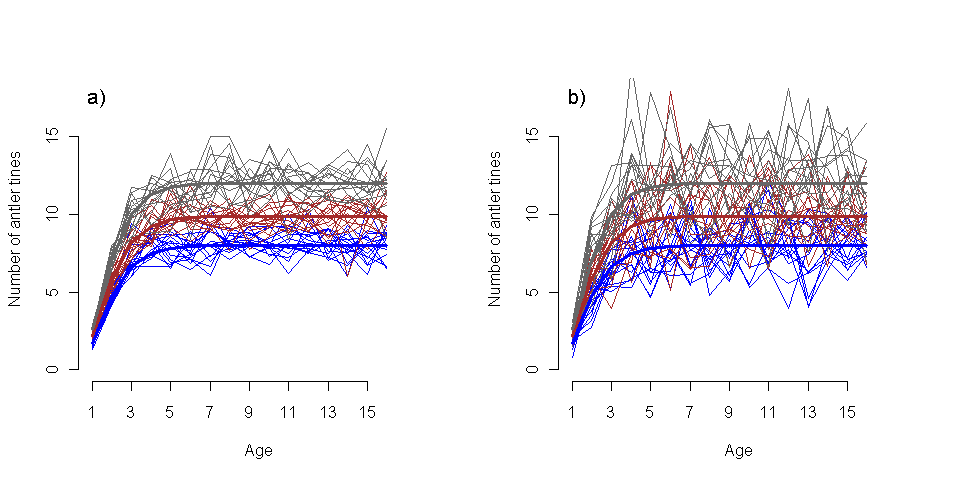


**Fig. S1.** Simulated sizes for individuals with 3 different antler size potentials (grey: 12 antler tines, red: average size asymptote in Norwegian red deer, blue: 8 antler tines) and 2 different levels of the annual errors in growth; a) SD = 10% of potential size at a given age, b) SD = 20% of potential size at a given age. Patterns of antler growth and variation therein correspond well with the development of red deer antlers on the international point scale (see figure 1 in Lockow 1991).


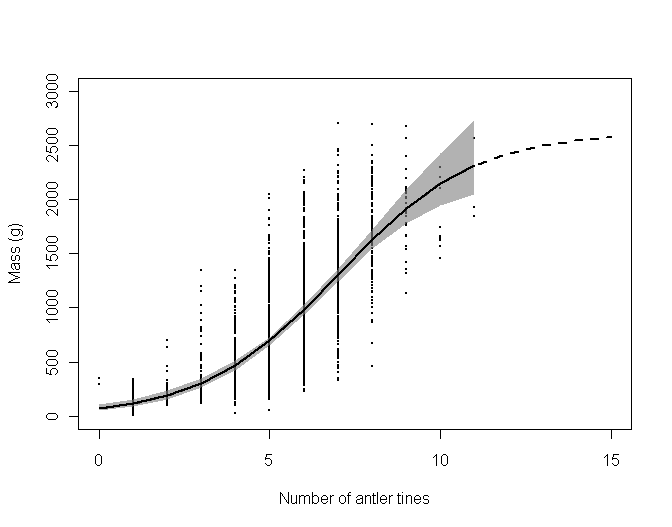


**Fig.** **S2.** Logistic function fit (solid black line) to antler mass and tine number data from Iberian red deer, kindly provided by Y. Fierro and summarized in Fierro et al. (2002). The hashed segment was the predicted antler mass associated with antler tine numbers ranging beyond the data. The grey area represents the 95% confidence band associated with the fit (not the data) and was based on the point-wise 0.025 and 0.975 quantiles of the predicted mass from 1000 bootstrap model fits (i.e., logistic function fitted to 1000 data sets re-sampled from the original data with replacement).


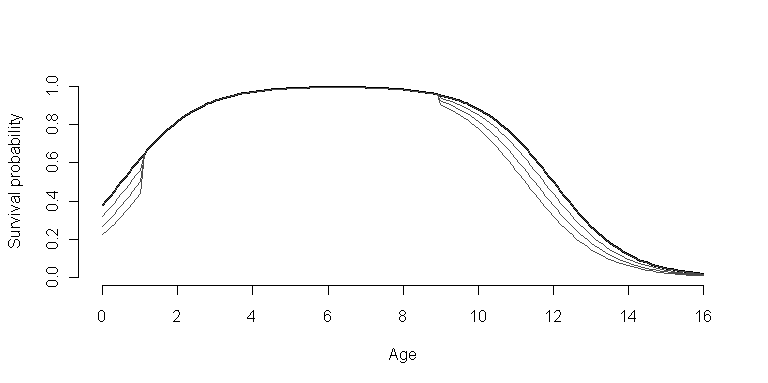


**Fig. S3**. Survival probability (natural) plotted as a function of age as used in the simulation model of male red deer. The function describing the curve (see main text) has been selected and parameterized to closely match age-specific survival probabilities reported for male red deer on the Isle of Rum by Catchpole *et al*. (2004). The thick black line represents age-specific survival in the absence of density dependent effects; thinner grey lines indicate density-dependent effects for young and senescent individuals (following empirical results from Catchpole *et al*. 2004), with density increasing from top to bottom: 0.5K, 1K, 1.5K (K =carrying capacity). Note that Catchpole *et al*. (2004) did not report values for calves (age = 0), while we included this.


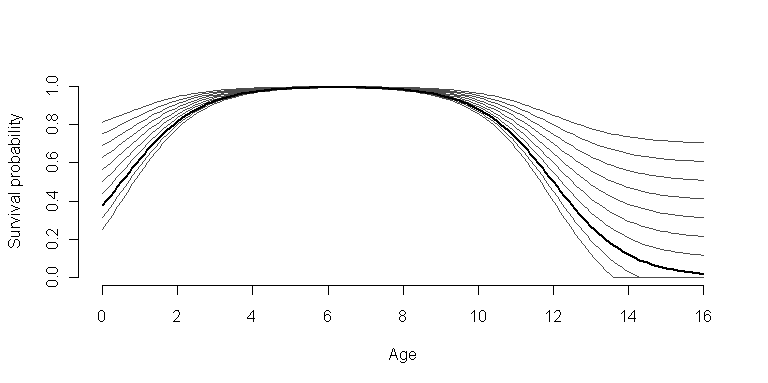


**Fig. S4.** Different levels of simulated age-specific survival probability (natural) of male red deer. The thick black line represents a risk multiplier of 1 (i.e. mortality magnitudes closely approximate those reported by Catchpole *et al*. (2004); thinner grey lines show the effect of multipliers ranging from 0.3 to 1.2 (from top to bottom in increments of 0.1).

VALIDATION

We found good correspondence between the dynamic behaviour of the simulated population and empirical reports of dynamic patterns. Heritability and correlation estimates from data simulated with the model were within the ranges reported for red deer in the literature. Additionally, model predicted distribution of antler size over ages under representative conditions, closely mimicked current age-size-distribution for Norwegian red deer. These and other evidence of correspondence with empirical results encourage confidence in model predictions, as does the fact that we limited forecast to the short- and intermediate-term (< 100 years). We add that a relatively small number of iterations (50) yielded clear response patterns, with mostly intuitive interpretations. Here we outline in more detail the validation of the individual-based simulation model.

1. Age structure
2. Antler growth and size distribution
3. Correlation between yearling and adult trait value

**V1. Age structure**

Our model resulted in a similar age structure and distribution of cohorts (Fig. S5) as has been documented empirically for red deer (figure 1c in Coulson *et al*. 2004), although with a somewhat higher average age (5 instead of 4 years) for individuals older than 12 months.


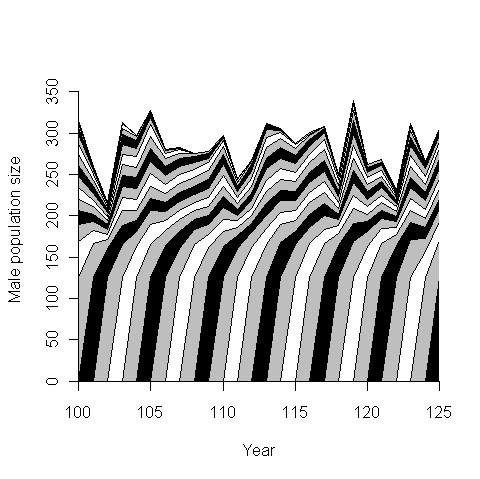


Fig. S5. Twenty-five year dynamics of a simulated population of male red deer without hunting. Each band (alternating black, grey, and white) represents a cohort. Individual cohort bands stack up to the total size of the population. The figure shows model-predicted patterns consistent with dynamics reported for red deer on the Isle of Rum (see Figure 1c in Couls*on et a*l. 2004). Evenly spaced lower portions of the bands were indicative of the steady birth rate from a fixed female population assumed in the model for simplicity.

**V2. Antler growth and size distribution**

The pattern of antler growth resulting from our simulations (Fig. S1) was very similar to the development of red deer antlers on the international point scale (see figure 1 in Lockow 1991). This includes annual antler scores which (i) can decline from one year to the next and (ii) have an error that was smaller for individuals that were on a lower or delayed growth trajectory, supporting our use of an error structure implemented as a percentage of age-specific potential size. The close approximation of the simulated distribution of sizes in the hunted population to Norwegian red deer data from a hunted population (Mysterud et al. 2005) was further indication of reasonable model fit with respect to antler development and size distribution (Fig. S6).


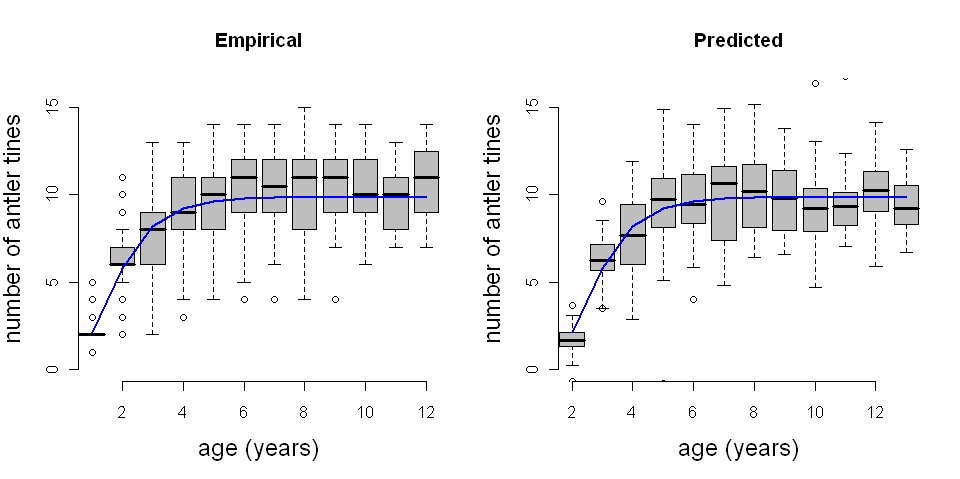


**Fig. S6.** Boxplots showing the distribution of antler sizes over all ages in Norwegian red deer (left) and a simulated population from the individual-based model. Both the real and the simulated population were hunted. The blue line represents the Gompertz growth curve, fitted to the Norwegian population.

**V3. Correlation between yearling and adult trait value**

We found that an annual individual error from a normal distribution with mean 0 and a standard deviation between 10% -15% of potential age-specific size (*ε_a_* in equation 1) resulted in correlation (Fig. S7) in simulated antler tine number in individuals at the yearling and 6-year-old stage that corresponds well with correlation estimates in empirical studies (r between 0.68-0.84; Schmi*dt et a*l. 2001; Bartos, Bahbouh & Vach 2007). Through the size of the annual error, we could conveniently manipulate yearling-to-adult correlation in antler size and explore model sensitivity to changes in this parameter.


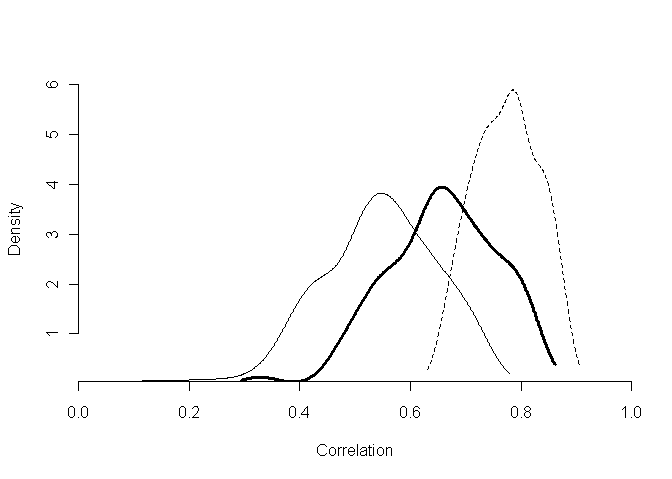


**Fig. S7.** Distribution of correlation estimates between simulated antler tine numbers of yearling and 6-year old red deer, when the annual error (*ε*) in antler growth was set to 0.1 (thin hashed line), 0.15 (thick solid line), and 0.2 (thin solid line). Estimates were calculated over the last 200 years of a 300-year simulation for each of the settings of *ε_a_* and with all other parameters at their default values.

**References**

Bartos, L., Bahbouh, R. & Vach, M. (2007) Repeatability of size and fluctuating asymmetry of antler characteristics in red deer (*Cervus elaphus*) during ontogeny. *Biological Journal of the Linnean Society*, **91,** 215-226.

Catchpole, E. A., Fan, Y., Morgan, B. J. T., Clutton-Brock, T. H. & Coulson, T. (2004) Sexual dimorphism, survival and dispersal in red deer. *Journal of Agricultural, Biological, and Environmental Statistics*, **9,** 1-26.

Coulson, T., Guinness, F., Pemberton, J. & Clutton-Brock, T. (2004) The demographic consequences of releasing a population of red deer from culling. *Ecology*, **85,** 411-422.

Fierro, Y., Gortazar, C., Landete-Castillejos, T., Vicente, J., Garcia, A. & Gallego, L. (2002) Baseline values for cast antlers of Iberian red deer (*Cervus elaphus hispanicus*). *Zeitschrift für Jagdwissenschaft*, **48,** 244-251.

Lockow, K.-W. (1991) Vorhersage der Geweihentwicklung des Rothirsches - eine Entscheidungshilfe für WahlabschuB und Hege. *Zeitschrift für Jagdwissenschaft*, **37,** 24-34.

Mysterud, A., Meisingset, E. L., Langvatn, R., Yoccoz, N. G. & Stenseth, N. C. (2005) Climate-dependent allocation of resources to secondary sexual traits in red deer. *Oikos*, **111,** 245-252.

Schmidt, K. T., Stien, A., Albon, S. D. & Guinness, F. E. (2001) Antler length of yearling red deer is determined by population density, weather and early life history. *Oecologia*, **127,** 191-197.
